# Letter to the editor: “Comment on: Application of the optimized carbon monoxide rebreathing method for the measurement of total haemoglobin mass in chronic liver disease”

**DOI:** 10.14814/phy2.14468

**Published:** 2020-05-29

**Authors:** Christoph Ahlgrim, Torben Pottgiesser

**Affiliations:** ^1^ Department of Cardiology and Angiology II Faculty of Medicine University Heart Center Freiburg‐Bad Krozingen Freiburg University Bad Krozingen Germany; ^2^ Department of Cardiology and Angiology I Faculty of Medicine University Heart Center Freiburg‐Bad Krozingen Freiburg University Freiburg Germany

Dear Editor,

With great interest, we have read the article “Application of the optimized carbon monoxide rebreathing method for the measurement of total hemoglobin mass in chronic liver disease“ by Plumb et al. (2020) and agree that CO‐rebreathing is a promising method to determine vascular volumes in clinical patients. However, we would like to point out that special attention should be paid to the distribution of the vascular volumes in patients with liver cirrhosis.

When calculating the total blood volume and thus the plasma volume using substances that mark erythrocytes or hemoglobin, the so‐called body/venous hematocrit ratio of ~0.9 is usually applied (Chaplin, Mollison, & Vetter, 1953).

Patients with liver cirrhosis and portal hypertension feature a pooling of blood in the splanchnic system (Busk et al., 2017), and their body/venous hematocrit ratio appears to be significantly lower (~0.8) (Lieberman & Reynolds, 1967). Moreover, the relative distribution of the blood is changed after establishing a portosystemic shunt (Busk et al., 2017).

The application of the correct body/venous hematocrit factor is crucial when calculating total blood volumes in patients with liver cirrhosis from erythrocyte‐ or hemoglobin‐based tracers (such as CO rebreathing), which has been highlighted before (Lieberman & Reynolds, 1967). This might be of special importance when longitudinal alterations of the blood volume shall be investigated using CO‐based techniques such as after introduction of a portosystemic shunt.

## CONFLICT OF INTEREST

All authors declare no conflict of interest.
